# Association between carbohydrate quality index and general and central obesity in adults: A population-based study in Iran

**DOI:** 10.34172/jcvtr.2021.47

**Published:** 2021-11-11

**Authors:** Nasim Janbozorgi, Kurosh Djafarian, Saba Mohammadpour, Mahtab Zareie Abyane, Mahdi Zameni, Mostafa Badeli, Zahra Akbarzade, Cain C. T. Clark, Sakineh Shab-Bidar

**Affiliations:** ^1^Department of Community Nutrition, School of Nutritional Sciences and Dietetics, Tehran University of Medical Sciences (TUMS), Tehran, Iran; ^2^Department of Clinical Nutrition, School of Nutritional Sciences and Dietetics, Tehran University of Medical Sciences (TUMS), Tehran, Iran; ^3^Centre for Intelligent Healthcare, Coventry University, Coventry, CV15FB, U.K

**Keywords:** Carbohydrate Quality, Obesity, Adults, Diet

## Abstract

***Introduction:*** To determine whether dietary carbohydrates quality index (CQI), glycemic index, and glycemic load is associated with general and abdominal obesity.

***Methods:*** 850 participants, 20 to 59 years old, were enrolled in a cross-sectional study from five Tehran districts through health houses. The 168 items in the semi--quantitative food frequency questionnaire were used to assess dietary intake. The CQI was calculated by using the following four components: glycemic index, total fiber, solid carbohydrate to total carbohydrate ratio, and whole grains: total grains ratio.

***Results:*** After adjusting for confounding factors, the chance of obesity in men (OR=0.38, 95% CI 0.15to 0.95; *P* =0.04) measured by waist circumference (WC) was significantly lower in the fourth quintile of CQI in comparison with the first quintile. In addition, OR for obesity in men (OR=2.53, 95% CI0.52 to 1.37; *P* =0.04) was significantly 2.5 times higher among those in the fourth quintile of glycemic index compared with those in the lowest quintile. There was no significant association between dietary carbohydrates with general obesity in men and women.

***Conclusion:*** In summary, dietary CQI is significantly inversely related to central obesity in men,according to this study. Additionally, adherence to a diet with a higher glycemic index in men is positively associated with central obesity.

## Introduction

Obesity represents one of the most prevalent and challenging topics in the healthcare systems in developing and developed countries.^1^ Increased adipose tissue in the body results in physiological dysfunction and increases the incidence of non-communicable diseases, such as diabetes mellitus, heart disease, some cancers, and skeletal muscle diseases.^2^ By 2030, two and a half billion adults are expected to be overweight or obese an increasing global trend of obesity.^1,3^ According to the survey of Non-Communicable Diseases risk factor surveillance in Iran, the national prevalence rates of overweight/obesity among Iranian adults was 59.3% in 2016.^4^ Aside from genetics, lifestyles and dietary habits can play an important role in obesity risk.^5-8^ Studies have shown that the risk of death from cardiovascular disease and fat deposition in the arteries of obese people is higher than in people with normal weight.^9^

Different strategies including specific diets to maintain optimal body weight in the long term have been suggested, however, the results of those strategies are controversial.^10^ Thus, one of the most prominent challenges in obesity research is identifying the role of macronutrients in the incidence of obesity.^10^ Nutrition- related diseases have been shown to be strongly linked to high carbohydrate diets.^11^

Previous attempts to investigate the influence of a quantitative carbohydrate index on metabolic syndrome components may be regarded as insufficient to discern disease causality. Thus, there is a need for a more comprehensive index to assess the overall quality of dietary carbohydrates.^12^ The carbohydrate quality index (CQI) consists of a variety of components, such as dietary fiber, glycemic index or glycemic load, whole grains: total grains ratio. and solid to total carbohydrate ratio.^13^ The correlation between overall carbohydrates quality and obesity was found in Spanish cohort study^12^. Moreover, another study showed an inverse relationship between overall CQI and general and abdominal obesity.^14^

Given the increasing prevalence of obesity in Iran and the cost of its complications, taking preventive measures must be adopted to improve quality of life and reduce early mortality. Given the high consumption of carbohydrates, especially white grains, in Iran, evaluation of the CQI index may provide insight for obesity management. Accordingly, the purpose of this study was to determine the correlation between carbohydrate intake quality and quantity for Iranian women and men with general and central obesity.

## Materials and Methods

### Participants

In this cross-sectional study, 850 adult males and females (20-59 years old) were recruited from health houses in different districts of Tehran, Iran. Data were collected from March 2017 to April 2019. The following formula was used for sample size calculation: n = (pqz2)/E2. Considering the total prevalence of 65% for overweight and obesity,^15^ an error coefficient of d = 0.04 and at a level of 0.05, the sample size of 546 people was obtained. With a design effect of 1.5 and to compensate for the potential exclusion of participants due to under- and over-reporting of total energy intake, or attrition due to other reasons, the final sample size of 850 participants was selected for inclusion. A two-stage cluster sampling was used to recruit participants from health care centers. Firstly, health houses were randomly selected, equitably spread across Tehran, Iran. Next, an equitable number of participants were randomly recruited from each health house. Samples were apparently healthy people who living in Tehran and people who met the following criteria: a) participants within the age range of 20-59 years; b) apparently healthy individuals who did not report any previous diagnosis of chronic diseases such as diabetes, cardiovascular diseases and chronic kidney, lung and liver diseases by a physician; c) be willing to take part in the study; d) being a resident of Tehran and e) being a member of the health center. Participants were excluded from the analysis if a) their daily energy intake was implausibly low or high (<800 Kcal/day or > 4200 Kcal/day); b) those who report any adherence to certain dietary patterns, any special diet or diet therapy such as vegetarian diet; and c) were migrant.

After excluding individuals who had at least one unfinished variable, 837 individuals were included in the final analysis. Participants gave written consent to participate in this project, which was supported Tehran University of Medical Sciences and carried out according to Helsinki declaration.

### Demographic factors

Information on age, sex (male, female), education (illiterate, under diploma, diploma, educated), marriage (single, married, divorced), smoking (never smoker, quit smoking, low smoking), and occupation (employee, housekeeper, retired, unemployed) was collected.

### Physical activity

International Physical Activity Questionnaire (IPAQ) were used assess physical activity. Metabolic Equivalents (METs) was used to categorize into very low (<600 MET-minute/week), low (600-3000 MET-minute/week), moderate and high ( > 3000 MET-minute/week).^16^

### Anthropometric and blood pressure assessment

Patient height was measured using a stadiometer with a sensitivity of 0.1 cm (Seca, Germany) under the condition that the participants were unshod, and their weight was measured by digital scales (808Seca, Germany), measured with 0.1 kg precision with light clothing (without coat and jacket). Body mass index (BMI) was calculated as weight in kg divided by squared height in meters squared. Waist circumference (WC) was recorded to the nearest 0.1 cm at the umbilical level, and hip circumference at the maximal level over light clothing, using a non-stretch tape measure, without putting pressure on the body surface. Blood pressure was measured by a digital barometer (BC 08, Beurer, Germany) after at least 10-15 minutes of rest and sitting, with the mean of two measurements being reported for each person.

### Dietary assessment and CQI calculation

We collected semi-quantitative data on food intake from food frequency questionnaire used in the past year. A 168 items questionnaire with reliable and validated results consists of a list of a list of food items with an estimate of the size of each item.^17^ In this survey, participants were asked how frequency they consume each food each day, week, month and annually. Kitchen utensils were used to indicate more precise amounts of food. The number of grams each person consumed was determined for each individual. The codes were then assigned to the memoirs and questionnaire information was entered into N4 software, and food intake was calculated based on the amount of food consumed. Participants’ dietary outcomes over the past year were used to determine the CQI index. The index includes four components: glycemic index, total fiber, solid carbohydrate to total carbohydrate ratio, and whole grains: total grains ratio. The glycemic index calculation method for each person was such that the food glycemic index was multiplied by the carbohydrate content of each food and then divided by the total daily carbohydrate content. Glycemic index food was obtained from a variety of methods, including international tables, the glycemic index of Iranian food book,^18,19^ and articles published in Iran. Glycemic load was calculated by multiplying the carbohydrate content consumed in each meal by the same glycemic index and then divided by 100, which is the considered desired glycemic load.^20^ Liquid carbohydrates were derived from the total carbohydrates of sweet drinks and juices consumed by the individual, and the remaining daily carbohydrates were considered solid carbohydrates. The CQI index was calculated based on the adjusted energy of the total amount of carbohydrate consumed through the residual method. For each of the CQI criteria, participants were divided into quintiles, with a score of one to five for each criterion. In Iran, this ratio was set to zero due to not using whole grains, and the overall CQI score ranged from 4 to 15.

### Definition of general and abdominal obesity

BMI greater than or equal to 30 kg/m2 was defined as general obesity.^21^ Women with waist circumference of ≥88cm and men with ≥102 cm waist circumference were considered abdominal obesity. A waist to hip ratio of ≥1 for the men and ≥ 0.85 for the women was taken into consideration when determining abdominal obesity.^22^

### Statistical methods

All data were analyzed using the statistical software package SPSS version 16 (SPSS version 16; SPSS Inc). We considered P < 0.05 to represent statistical significance level. In the CQI of the adjusted energy diet, participants were divided into quintiles. In CQI quintiles, quantitative variables are reported as mean and standard deviation and qualitative variables as frequency (percent) as tables. Chi-square test was used to compare the frequency of qualitative variables among the CQI quintiles and Analysis of Variance (ANOVA) was used to compare the means of quantitative variables. Also, nutritionist 4 software, adapted to Iranian foods, was used to analyze nutrients intake. To diagnose abdominal obesity in participants, Odds Ratios (OR) and Confidence Intervals (CI) were determined through binary logistic regression in two target models; unadjusted, and adjusted for BMI, physical activity, age, sex, energy, education, and marital status.

## Results

At the beginning of the study, 850 patients were enrolled, 13 of whom were excluded from the study due to their unwillingness to complete questionnaire, resulting in 837 participants completing the study. The mean age and BMI of participants were 44.77 ± 10.6 years and 27.89 ± 5.56 kg/m2, respectively. Also, mean waist circumference of men and women was 95.51 ± 12.02 and 90.55 ± 12.30 cm, respectively. As well, mean CQI score of males and females were 8.68 ± 2.58, 9.15 ± 2.56, respectively.

As detailed in Table 1, there was no significant difference between the general characteristics of participants across quintiles of CQI. Although the mean of weight, WC, and BMI increased, there were no significant differences in anthropometric measures and indices across quintiles of CQI.

**Table 1:** General characteristics, anthropometric measures and indices of participants according to quintiles (q) of carbohydrate quality index (CQI)

**Characteristics**		**CQI**					***P*** ** value**
		**Q1 (n=136)**	**Q2 (n=218)**	**Q3 (n=115)**	**Q4 (n=223)**	**Q5 (n=145)**	
		Mean ± SD					
Age(years)		44.7 ± 10.5	44.3 ± 10.9	46.8 ± 10.1	44.0 ± 10.8	44.7 ± 10.7	0.22
Weight(kg)		73.4 ± 14.3	73.8 ± 12.3	74.7 ± 14.4	72.4 ± 13.1	74.0 ± 14.6	0.62
Height (cm)		163 ± 9.10	163 ± 8.75	162 ± 8.63	162 ± 8.63	162.2 ± 9.50	0.37
BMI (kg/m^2^)		27.4 ± 4.56	27.6 ± 4.13	28.8 ± 9.07	27.7 ± 5.30	28.4 ± 4.97	0.24
WC (cm)		92.2 ± 12.2	92.5 ± 12.1	93.3 ± 11.6	90.6 ± 12.1	93.2 ± 13.1	0.20
Hip (cm)		103 ± 8.64	104 ± 11.1	105 ± 11.7	103 ± 10.9	105 ± 10.99	0.22
WHR(cm)		0.89 ± 0.08	0.88 ± 0.08	0.90 ± 0.24	0.87 ± 0.07	0.88 ± 0.09	0.38
		**N (%)**					
HTN n(%)	Yes	22(16.8)	35(26.7)	11(8.4)	42(32.1)	21(16)	0.22
	No	114(16.1)	183(25.9)	104(14.7)	181(25.5)	124(17.5)	
Sex n(%)	Male	49(18.8)	74(28.4)	34(13)	63(24.1)	41(15.7)	0.42
	Female	87(15.1)	144(25)	81(14.1)	160(27.8)	104(18.1)	
Education n(%)	Illiterate	13(18.1)	17(23.6)	17(23.6)	16(22.2)	9(12.5)	0.11
	Under diploma	44(19.9)	55(24.9)	31(14)	60(27.1)	31(14)	
	Diploma	31(12.1)	66(25.8)	34(13.3)	78(30.5)	47(18.4)	
	Educated	48(16.7)	80(27.8)	33(11.5)	69(24)	58(20.1)	
Occupation n(%)	Employee	36(16.8)	58(27.1)	27(12.6)	51(23.8)	42(19.6)	0.84
	Housekeeper	73(15.5)	122(25.8)	69(14.6)	131(27.8)	77(16.3)	
	Retired	25(19.8)	33(26.2)	14(11.1)	34(27)	20(15.9)	
	Unemployed	2(8)	5(20)	5(20)	7(28)	6(24)	
Activity score n(%)	Low	86(16.1)	151(28.3)	72(13.5)	140(26.3)	84(15.8)	0.27
	Moderate	50(16.4)	67(22)	43(14.1)	83(27.3)	61(20.1)	
Menopause n(%)	Yes	36(13.1)	61(22.3)	44(16.1)	77(28.1)	56(20.4)	0.06
	No	100(17.8)	157(27.9)	71(12.6)	146(25.9)	89(15.8)	
Marriage n(%)	Single	18(20.2)	24(27)	10(11.2)	24(27)	13(14.6)	0.92
	Married	107(15.7)	179(26.3)	93(13.7)	180(26.5)	121(17.8)	
	Divorce	11(16.2)	15(22.1)	12(17.6)	19(27.9)	11(16.2)	
Smoking n(%)	Not smoking	122(16.1)	198(26.1)	105(13.8)	204(26.9)	130(17.1)	0.5
	Quit smoking	7(20.6)	7(20.6)	5(14.7)	5(14.7)	10(29.4)	
	Low smoking	7(15.9)	13(29.5)	5(11.4)	14(31.8)	5(11.4)	

Abbreviations: BMI,body mass index; WC, waist circumference; WHR, waist to hip ratio **Participants were divided into categories called quintiles. *** Carbohydrate quality index including total fiber, glycemic index, whole grains: total grains ratio, solid carbohydrate to total carbohydrate ratio carbohydrate quality score range: Q1: 4–8, Q2: 9–10, Q3: 11–12, Q4: 13–14, Q5: 15–20). ***Quantitative and qualitative variable obtained from one-way ANOVA, Chi-Square test, respectively.

Table 2 shows dietary intake based on quintiles of CQI, and there was a significant difference between quintiles in the dietary glycemic index (*P* < 0.001), carbohydrate intake (*P* = 0.01), solid carbohydrates (*P* = 0.002), liquid carbohydrates (*P* < 0.001), total fiber intake (*P* < 0.001), refined grains (*P* < 0.001), and dietary glycemic load (*P* < 0.001).

**Table 2:** Dietary intake and indices based on quintiles (q) of carbohydrate quality index criteria (CQI)

	**CQI**					***P*** ** value**
	**Q1 (n=136)**	**Q2 (n=218)**	**Q3 (n=115)**	**Q4 (n=223)**	**Q5 (n=145)**	
Dietary glycemic index	63.3 ± 29.0	46.2 ± 26.2	37.3 ± 19.8	33.3 ± 34.7	24.4 ± 8.8	<0.001
Carbohydrate intake (g/day)	338 ± 129	375 ± 165	368 ± 145	365 ± 170	411 ± 241	0.01
Solid carbohydrates (g/day)	301 ± 127	348 ± 161	347 ± 139	343 ± 165	383 ± 232	0.002
Liquid carbohydrates (g/day)	13.8 ± 33.0	8.83 ± 14.48	3.46 ± 4.07	2.45 ± 4.94	1.08 ± 1.40	<0.001
Total fiber intake(g/day)	13.3 ± 5.24	17.8 ± 11.1	17.6 ± 8.8	20.0 ± 12.5	26.1 ± 11.7	<0.001
Refined grains (g/day)	215 ± 132	185 ± 119	161 ± 90.9	139 ± 85.9	115 ± 61.9	0.001
Dietary glycemic load (g/day)	307 ± 124	231 ± 129	184 ± 93	162 ± 107	127 ± 44	<0.001

*Values are mean ± standard deviation for variables. **Participants were divided into categories called quintiles. *** Carbohydrate quality index including total fiber, glycemic index, whole grains: total grains ratio, solid carbohydrate to total carbohydrate ratio carbohydrate quality score range: Q1: 4–8, Q2: 9–10, Q3: 11–12, Q4: 13–14, Q5: 15–20). *** Obtained from one-way ANOVA.

Logistic regression analysis [odds ratio (OR) 95% confidence interval (CI)] of abdominal and general obesity across categories of CQI is shown in Table 3. There was a significant relationship between CQI quintile with abdominal obesity based on WC definition in crude (*P* = 0.04) and adjusted model (*P* = 0.04) in men. Those in the fourth CQI quintile had a lower chance of abdominal obesity compared to those in the first CQI quintile in men both in crude (OR 0.39, 95%CI 0.55 to 1.29; for trend, *P* = 0.04) and in the adjusted model (OR 0.38, 95%CI 0.15 to 0.95; for trend, *P* = 0.04). We did not find any significant association between adherence to CQI and other adiposity measures.

**Table 3:** Logistic regression analysis [odds ratio (OR) 95% confidence interval (CI)] of obesity according to quintiles (q) of carbohydrate quality index (CQI)

	**Q1** **(n=136)**	**Q2** **(n=218)**		**Q3** **(n=115)**		**Q4** **(n=223)**		**Q5** **(n=145)**	
	**Ref.**	**OR**	**95% CI**	**OR**	**95% CI**	**OR**	**95% CI**	**OR**	**95%CI**
WC									
Women									
Model1	1	0.96	0.56,1.65	1.31	0.71,2.44	0.94	0.56,1.60	1.29	0.72,2.31
*P* value		0.91		0.38		0.84		0.38	
Model2	1	0.96	0.52,1.76	1.02	0.51,2.05	0.87	0.48,1.57	1.12	0.58,2.17
*P* value		0.90		0.94		0.66		0.72	
Men									
Model1	1	1.02	0.65,1.54	0.90	0.75,2.04	0.39	0.55,1.29	0.77	0.75,1.91
*P* value		0.96		0.82		0.04		0.58	
Model2	1	0.89	0.40,1.99	0.86	0.33,2.25	0.38	0.15,0.95	0.81	0.31,2.11
*P* value		0.79		0.76		0.04		0.68	
WHR									
Women									
Model1	1	0.91	0.53,1.56	0.92	0.50,1.69	1.07	0.63,1.82	1.05	0.59,1.88
*P* value		0.75		0.79		0.78		0.84	
Model2	1	0.93	0.53,1.65	0.79	0.41,1.51	1.05	0.60,1.85	0.97	0.52,1.80
*P* value		0.81		0.47		0.84		0.94	
Men									
Model1	1	1.89	0.81,4.38	1.70	0.60,4.76	0.82	0.37,1.82	1.36	0.53,3.48
*P* value		0.13		0.31		0.63		0.51	
Model2	1	1.71	0.70,4.22	1.62	0.54,4.84	0.85	0.36,2.01	1.65	0.60,4.52
P value		0.23		0.38		0.71		0.33	
BMI									
Women									
Model1	1	0.67	0.36,1.26	0.72	0.42,1.25	1.36	0.75,2.48	0.81	0.47,1.37
*P* value		0.22		0.25		0.30		0.43	
Model2	1	1.11	0.59,2.10	1.82	0.91,3.60	1.18	0.63,2.18	1.45	0.75,2.81
*P* value		0.72		0.08		0.59		0.26	
Men									
Model1	1	1.24	0.48,3.18	0.60	0.23,1.54	1.69	0.62,4.60	1.05	0.42,2.62
*P* value		0.65		0.28		0.30		0.90	
Model2	1	0.10	0.19,1.15	1.35	0.52,3.49	0.81	0.34,1.91	0.75	0.28,2.005
*P* value		0.47		0.52		0.63		0.57	

Abbreviations: BMI, body mass index; WC, waist circumference; WHR: waist to hip ratio *Participants were divided into categories called quintiles. **Carbohydrate quality index including total fiber, glycemic index, whole grains: total grains ratio, solid carbohydrate to total carbohydrate ratio carbohydrate quality score range: Q1: 4–8, Q2: 9–10, Q3: 11–12, Q4: 13–14, Q5: 15–20). *** Central obesity: waist circumference men≥102 cm, women≥88cm, Waist to hip ratio men≥1 cm, women ≥ 0.85 cm, BMI > 30 kg/m^2^. Model 1: crude; Model 2: adjusted for age, menopause, marriage, smoking, occupation, education, activity score, total energy.

Logistic regression analysis [odds ratio (OR) 95% confidence interval (CI)] of abdominal and general obesity across categories of the dietary glycemic index is shown in Table 4. There was significant relationship between fourth dietary glycemic index quintile and abdominal obesity measured by WC in crude (*P* = 0.02) and adjusted model (*P* = 0.04) in men, even after controlling for confounders in which those with higher dietary glycemic index had a lower probability of central obesity. However, we did not observe any significant relationship between dietary glycemic index and central obesity measured in men.

**Table 4:** Logistic regression analysis [odds ratio (OR) 95% confidence interval (CI)] of abdominal and general obesity according to quintiles (q) of glycemic index

	**Q1**	**Q2**		**Q3**		**Q4**		**Q5**	
	**Ref**	**OR**	**95% CI**	**OR**	**95% CI**	**OR**	**95% CI**	**OR**	**95%CI**
WC									
Women									
Model1	1	1.11	0.66,1.86	0.59	0.35,0.98	0.96	0.57,1.63	1.22	0.47,1.39
P value		0.69		0.04		0.90		0.46	
Model2	1	1.39	0.77,2.48	0.62	0.34,1.11	0.98	0.54,1.77	1.60	0.71,2.08
P value		0.26		0.11		0.96		0.12	
Men									
Model1	1	1.56	0.59,4.12	1.55	0.60,4.02	2.81	1.13,6.95	2.00	0.80,4.97
P value		0.36		0.36		0.02		0.13	
Model2	1	1.15	0.41,3.19	1.47	0.55,3.88	2.53	1.004,6.42	1.68	0.66,4.29
P value		0.78		0.43		0.04		0.27	
WHR									
Women									
Mode1	1	0.81	0.48,1.36	0.72	0.43,1.22	0.90	0.53,1.52	0.86	0.51,1.46
P value		0.43		0.22		0.70		0.59	
Mode2	1	0.87	0.50,1.50	0.80	0.46,1.39	0.92	0.52,1.60	0.94	0.53,1.64
P value		0.62		0.43		0.76		0.83	
Men									
Model1	1	1.14	0.46,2.78	1.15	0.48,2.76	1.69	0.68,4.21	1.14	0.48,2.66
P value		0.77		0.75		0.25		0.76	
Model2	1	0.76	0.29,1.59	1.05	0.42,2.63	1.47	0.56,3.85	0.92	0.37,2.26
P value		0.58		0.91		0.42		0.86	
BMI									
Women									
Model1	1	0.96	0.55,1.71	0.83	0.47,1.48	0.97	0.55,1.68	0.99	0.57,1.75
P value		0.89		0.52		0.91		0.98	
Model2	1	1.08	0.61,1.92	0.91	0.50,1.64	1.01	0.56,1.81	1.08	0.60,1.95
P value		0.78		0.76		0.95		0.78	
Men									
Model1	1	1.81	0.67,4.88	1.79	0.67,4.77	1.66	0.62,4.40	1.66	0.63,4.34
P value		0.24		0.24		0.30		0.30	
Model2	1	1.99	0.72,5.52	1.77	0.66,4.72	1.71	0.63,4.57	1.72	0.65,4.55
P value		0.18		0.25		0.28		0.27	

Abbreviations: BMI, body mass index; WC, waist circumference; WHR, waist to hip ratio *Participants were divided into categories called quintiles. **Carbohydrate quality index including total fiber, glycemic index, whole grains: total grains ratio, solid carbohydrate to total carbohydrate ratio carbohydrate quality score range: Q1: 4–8, Q2: 9–10, Q3: 11–12, Q4: 13–14, Q5: 15–20). Model 1: crude; Model 2: adjusted for age, menopause, marriage, smoking, occupation, education, activity score, total energy. BMI, body mass index; WC, waist circumference; WHR, waist-to-hip ratio. General obesity by BMI: 30 and above (kg/m2); (yes/no), abdominal obesity measures: men (≥102 cm) and women (≥88cm; (yes/no), high WHR ≥1.18; (yes/no).

Unadjusted and adjusted OR and 95% CI of abdominal and general obesity across quintiles of dietary glycemic load are provided in Table 5. We did not find any significant association between adherence to dietary glycemic load and general and central obesity in both sexes.

**Table 5:** Logistic regression analysis [odds ratio (OR) 95% confidence interval (CI)] of abdominal and general obesity according to quintiles (q) of Glycemic Load

	**Q1**	**Q2**		**Q3**		**Q4**		**Q5**	
	**Ref**	**OR**	**95% CI**	**OR**	**95% CI**	**OR**	**95% CI**	**OR**	**95%CI**
WC									
Women									
Model 1	1	0.82	0.49,1.37	0.64	0.38,1.07	0.92	0.54,1.56	1.01	0.59,1.75
*P* value		0.45		0.09		0.77		0.94	
Model 2	1	0.99	0.55,1.76	0.65	0.36,1.16	1.00	0.56,1.80	1.20	0.65,2.33
*P* value		0.98		0.14		0.98		0.54	
Men									
Model 1	1	1.43	0.53,3.87	2.29	0.90,5.80	2.42	0.96,6.10	2.22	0.91,5.38
*P* value		0.47		0.08		0.06		0.07	
Model 2	1	1.20	0.43,3.36	2.10	0.80,5.48	2.20	0.84,5.75	2.13	0.85,5.53
*P* value		0.72		0.12		0.10		0.10	
Women									
WHR									
Model 1	1	0.79	0.47,1.32	0.66	0.39,1.11	0.95	0.56,1.66	0.82	0.48,1.41
*P* value		0.38		0.12		0.87		0.48	
Model 2	1	0.93	0.54,1.61	0.73	0.42,1.26	1.02	0.53,1.77	0.89	0.50,1.57
*P* value		0.81		0.26		0.93		0.69	
Men									
WHR									
Model 1	1	1.10	0.44,2.77	1.41	0.57,3.53	1.16	0.48,2.82	1.06	0.46,2.43
*P* value		0.83		0.45		0.72		0.78	
Model 2	1	0.85	0.32,2.26	1.20	0.46,3.14	1.02	0.39,2.62	0.98	0.41,2.34
*P* value		0.75		0.70		0.96		0.96	
Women									
BMI									
Model 1	1	0.89	0.51,1.53	0.91	0.52,1.57	1.12	0.65,1.92	0.77	0.43,1.38
*P* value		0.68		0.74		0.67		0.39	
Model 2	1	0.98	0.54,1.75	0.94	0.52,1.68	1.22	0.69,2.16	0.77	0.41,1.42
*P* value		0.98		0.83		0.49		0.40	
Men									
BMI									
Model 1	1	1.46	0.52,4.11	2.41	0.92,6.31	1.57	0.58,4.27	1.92	0.75,4.87
*P* value		0.46		0.07		0.37		0.16	
Model 2	1	1.49	0.52,4.25	2.44	0.92,6.67	1.62	0.59,4.48	1.96	0.76,5.03
*P* value		0.45		0.07		0.34		0.16	

Abbreviations: BMI, body mass index; WC, waist circumference; WH, waist to hip ratio *Participants were divided into categories called quintiles. **Carbohydrate quality index including total fiber, glycemic index, whole grains: total grains ratio, solid carbohydrate to total carbohydrate ratio carbohydrate quality score range: Q1: 4–8, Q2: 9–10, Q3: 11–12, Q4: 13–14, Q5: 15–20). Model 1: crude; Model 2: adjusted for age, menopause, marriage, smoking, occupation, education, activity score, total energy. General obesity by BMI: 30 and above (kg/m2); (yes/no), abdominal obesity measures: men (≥102 cm) and women (≥88cm; (yes/no), high WHR ≥1.18; (yes/no).

## Discussion

The findings of our study suggest that, after adjustment for potential confounders, the consumption of diets with high CQI is inversely associated with abdominal obesity measured by WC in men. Moreover, the risk of abdominal obesity increased with greater adherence to a high glycemic index in men. None of the dietary carbohydrate indexes was associated with general and central obesity in women.

Previous studies have shown that, in the Iranian population, more than 60 percent of the total energy consumed comes from carbohydrates, especially refined carbohydrates with a high glycemic index.^23,24^ Moreover, in recent decades the intake of simple sugar has increased, whilst fruits and vegetable consumption has reduced.^25^ However, there is a paucity of data on the association between glycemic index, glycemic load, total fiber, and carbohydrate intake, including solid carbohydrates, with central and general obesity.

Only a limited number of studies^13,26-28^ used the CQI index, which is a combination of the components listed above in a single score. Results of the studies showed that CQI is associated with an increased risk of cardiovascular diseases, such as hypertension, insulin resistance, and obesity.^12,29,30^

An animal-based study^31^ demonstrated that carbohydrate intake, including the polysaccharide, guar gum, was not significantly associated with obesity, whereas another study in animals showed that obesity could be prevented by reducing carbohydrate intake.^32^ Concordant with our findings, other studies originating from Iran also reported that there was no significant relationship between carbohydrate intake and the odds of obesity,^33^ nor between low carbohydrate score and abdominal obesity.^34^ On the other hand, a significant relationship between the CQI index and central and general obesity in female Ghanaian women 14, and in a Korean population.^35^ However, in two recent clinical trials in obese subjects, carbohydrate quality and quantity had no significant influence on weight loss.^36,37^ In contrast, Saslow et al found that, after twelve weeks of a very low-carbohydrate diet, obese adults had significant weight loss.^38^ Findings of a recently published meta-analysis showed that there was no significant relationship between carbohydrate intake or increased percentage of total energy intake and the chance of obesity.^39^ Whilst, in another meta-analysis, low carbohydrate diets were associated with greater improvements in weight loss, compared to low-fat diets, in studies ranging from 8 weeks to 24 months in duration.^40,41^ Moreover, according to the results of another meta-analysis, low carbohydrate diets elicited beneficial effects on body weight in the short term. One conceivable reason for the discrepancies between the results of studies may be related to the differences in the study population, small sample sizes, and varying demographic factors such as age, race, and gender of participants, in addition to the use of different definitions of obesity.

In the present study, we also found that CQI components, such as glycemic index, were significantly associated with abdominal obesity. Accordingly, animal studies have shown that fat mass and obesity-associated (FTO) gene expression increases with increasing consumption of foods with high glycemic index content.^42^ Consistently, two human studies showed that high CQI levels may be associated with a lower chance of abdominal obesity.^14,35^ On the other hand, Fosini et al showed that there was no significant relationship between glycemic index and anthropometric factors, such as waist circumference and body mass index, among apparently healthy Iranian adults^43^ and Danish men.^44^ An intervention study showed that consumption of foods with a lower glycemic index led to more weight loss,^45^ however, Robert et al showed that participants receiving low glycemic index foods did not elicit significant decreases in body fat mass.^36^ Huffman et al reported that foods with a low glycemic index may have a beneficial effect on obesity and overweight.^46^ In addition, pooling studies in a meta-analysis suggested that there was no significant relationship between glycemic load and metabolic syndrome components, including waist circumference.^47^ One reason for the inconsistency in the studies is the amount and variety of carbohydrates consumed, especially rice, which is the dominant food in Asia.^48,49^ The proportion of total energy intake from carbohydrates in the Iranian population has been estimated to be 65 which mostly comes from rice and bread (49.8%).^50,51^ In comparison to other countries, the glycemic index and load in our population were lower which may be partially related to the different foods item consumed in Asia^50,52^ versus Western countries.^53-55^ Previous studies showed that potatoes, breakfast cereals, bread, and rice are major determinants of glycemic index and load in Western populations.^54^ These different findings may be partially due to differences in study design, target population, and dietary assessment tools used to determine dietary glycemic index and glycemic load. Another reason for the lack of agreement between studies may be attributed to cultural differences of the studied populations, including dietary habits and lifestyle, and evaluation of dietary habits in studies with different methods.

Some possible mechanisms for the association between CQI components and the chance of obesity have been suggested. Indeed, one of the mechanisms proposed is that the glycemic index can affect blood glucose fluctuations by consuming foods with high glycemic index content, reducing blood glucose fluctuations, and leading to increased appetite and energy intake; whilst for foods with a low glycemic index, fluctuations in blood sugar increase, leading to a decrease in appetite and ultimately decreasing energy intake.^56-62^ Another mechanism that has been suggested is that hyperinsulinemia results from the consumption of high-glycemic-index foods, which, in turn, results in reduced fat oxidation and increased carbohydrate oxidation, leading to an increase in fat storage of individuals.^60,61^ On the other hand, dietary fiber regulates body weight through a variety of mechanisms, including improved insulin sensitivity, reduced hunger, increased satiety, delayed intestinal transit, and regulation of lipid and sugar oxidation.^63-67^ Consumption of various forms of carbohydrates, including liquid and solid, reportedly increases the risk of obesity.^68-71^ Liquid carbohydrates, especially sweetened beverages that have a high glycemic index, increase the risk of obesity compared to solid carbohydrates in a variety of ways, including increased appetite, increased postprandial blood glucose levels, and reduced insulin sensitivity.^72,73^ Consumption of whole grains, insulin responses, and blood sugar is reduced by slowing digestion and absorption of starch; also reducing energy consumption by increasing satiety and decreasing appetite, potentially preventing or ameliorating obesity through these mechanisms.^63,74^

One of the strengths of this study is that, to the author’s knowledge, this is the first study to have examined the relationship between quality of carbohydrate intake (CQI) and abdominal obesity in Iran, which can be used as a guide for future studies in this field in Iran. Additionally, the large sample size of this study compared to other studies is an important advantage, meaning we can assert greater generalizability to the target population.

Despite the strengths of the novel study, some limitations must be considered. Firstly, participants had a CQI score of 15 due to not consuming whole grains, in addition, although a validated food frequency questionnaire was used to record the participants’ annual food intake, foods reported annually were more likely to be over-or under-reported. Another limitation of this study is the cross-sectional design, which prevents definitive certainty of these results. In addition, this study was implemented in winter, thus, low fruit variety in this season, combined with lack of memory recall for fruit consumption in other seasons may be a factor in low reporting rates.

## Conclusion

As a result of the current study, there has been no significant relationship between dietary CQI with central obesity in men. Additionally, adherence to a diet with a higher glycemic index in men is positively associated with central obesity.

## Acknowledgements

The authors wish to thank the subjects for their participation in the study.

## Competing interests

All of authors declared that they have no competing interests.

## Ethical approval

The Ethics Committee of Tehran of Tehran University approved all procedures involving research study participants (Ethics Number: IR.TUMS.VCR.REC.1397.157). All procedures for the study were in accordance with Helsinki declaration guidelines.

## Funding

This manuscript has been granted by the Tehran University of Medical Sciences (Grant No: 40186).
